# Quantification of adhesion of mesenchymal stem cells spread on decellularized vein scaffold

**DOI:** 10.1590/ACB361001

**Published:** 2021-11-05

**Authors:** Lenize da Silva Rodrigues, Ana Lívia de Carvalho Bovolato, Bárbara Esteves Silva, Leticia Victória Chizzolini, Bianca Latance da Cruz, Marcelo Padovani de Toledo Moraes, Pedro Luiz Toledo de Arruda Lourenção, Matheus Bertanha

**Affiliations:** 1 Fellow PhD degree. Postgraduate Program in Surgery and Translational Medicine - Department of Surgery and Orthopedics - Botucatu Medical School - Universidade Estadual Paulista (UNESP) – Botucatu (SP), Brazil.; 2 Fellow PhD degree. Postgraduate Program in Biotechnology - Botucatu Institute of Biosciences - Cell Engineering Laboratory - Botucatu Medical School - Universidade Estadual Paulista (UNESP) – Botucatu (SP), Brazil.; 3 Graduate student. Botucatu Medical School - Universidade Estadual Paulista (UNESP) – Botucatu (SP), Brazil.; 4 PhD. Department of Pathology - Botucatu Medical School - Universidade Estadual Paulista (UNESP) – Botucatu (SP), Brazil.; 5 PhD, Assistant Professor. Department of Surgery and Orthopedics - Botucatu Medical School - Universidade Estadual Paulista (UNESP) – Botucatu (SP), Brazil.; 6 PhD, Assistant Professor. Department of Surgery and Orthopedics - Botucatu Medical School - Universidade Estadual Paulista (UNESP) – Botucatu (SP), Brazil.

**Keywords:** Mesenchymal Stem Cells, Endothelium, Blood Vessels, Peripheral Arterial Disease

## Abstract

**Purpose:**

To evaluate methods that improve adipose-derived stem cells (ASCs) population in decellularized biological venous scaffold for tissue engineering in blood vessels, a model in rabbits.

**Methods:**

The ASC was expanded until the third passage. Inferior vena cava (IVC) was submitted to the decellularization process using 1% sodium dodecyl sulfate (SDS) or 2% sodium deoxycholate (SD) to compose 12 study groups (G): pure SD or SDS, exposed or not to 1% TritonX-100 (TX-100) and exposed or not to poly-l’lysine and laminin (PL). Scaffolds were covered with 1 × 10^5^ or 1 × 10^6^ ASCs diluted in 10 μL Puramatrix™. The histological analysis was done by cell counting in hematoxylin and eosin (HE) and nuclei count in immunofluorescence (IF) with 4’,6-Diamidine-2’-phenylindole dihydrochloride (DAPI).

**Results:**

The study of groups in HE and IF showed similar results. For both analyses,IVC-SD-1 × 10^6^ ASC and IVC-SD-PL-1 × 10^6^ ASC provided the best results. The IF technique showed better sensitivity than HE, with a weak agreement between them.

**Conclusions:**

Decellularizing agent and the number of ASC influence scaffolds cellularization response and the best protocols as those ones using SD with or without the addition of PL.

## Introduction

The tissue engineering of blood vessels (TEBV) represents a promising perspective of vascular substitutes in revascularization surgeries, such as coronary or peripheral arterial diseases. Many strategies to optimize the mechanisms of blood vessel production were investigated, considering specific needs to meet a demand for personalized medicine, in addition to pay attention to the need for biocompatibility^1^^-^ 3.

On the other hand, concerning the scaffolds, additional factors must be considered, such as the maintenance of a three-dimensional (3D) structure, tissue permeability, sufficient strength to resist blood pressure, elasticity, high durability, facilitation of attachment, migration, proliferation, and cell interaction with adjacent tissues. Additionally, this material should maintain the antithrombotic activity and provide a microenvironment that imitates the natural architecture of in-vivo tissue for the seeded cells^3^^-^ 6. The adipose tissue (AT) is an abundant source of adipose-derived stem cells (ASCs), easy to obtain in surgery. The discovery of techniques that promote tissue differentiation, with high exhibitions of plasticity, shows that the cultivation of ASCs can contribute a great deal to TEBV^7^^-^ 12.

Among the alternatives to produce scaffolds, there is the use of decellularized tissue-derived scaffolds^9^^,^ 10. The advantages of this material are related to the essential elements contained in the organic extracellular matrix, such as collagen, elastin, proteoglycans, cell adhesion proteins, glycosaminoglycans (GAGs), which are strong promoters of cellular adhesion and differentiation. Previous experimental studies conducted by this research group have demonstrated that two protocols for decellularization of rabbit’s vena cava were effective in cell removal and maintenance of the biomechanical characteristics of the 3D scaffold^13^^,^ 14.

Although many protocols for decellularization of organs and tissues are described, including the decellularization of blood vessels, with various decellularization agents and times^15^^,^ 16 for rabbit’s inferior vena cava, under a rapid decellularization method, our research team’s best results were obtained using 1% sodium dodecyl sulfate (SDS) and 2% sodium deoxycholate (SD)^13^^,^ 14 ^,^ 17 ^-^ 19.

Therefore, this study aimed to evaluate strategies that improve the number of ASC in these scaffolds as a model for future use in TEBV.

## Methods

### Animal housing conditions and tissue harvesting

The inferior vena cava (IVC) and the adipose tissue (AT) were harvested from 12 non-pregnant female adult rabbits (New Zealand). After the Committee on Animal Research and Ethics (CEUA) approval (Process no. 1279/2018), all procedures were conducted respecting the Ethical Guidelines for Animal Experimentation from Brazilian College for Animal Experimentation and were conducted following the U.S. National Institutes of Health or European Commission guidelines.

Animals were housed under controlled conditions and fed a standard pellet diet with water *ad libitum*. The median age and weight were 3 months and approximately 2.5 kg, respectively. Before the surgical procedures to obtain the AT and IVC, animals were anesthetized with tiletamine hydrochloride/zolazepam hydrochloride (20 mg/kg, i.m.) associated with 2% xylazine chloride (4 mg/kg, i.m.). The harvest was conducted under rigorous aseptic conditions, and, at the end of the procedure, animals were euthanized with pentobarbital.

### Scaffold production

The veins were fragmented in three segments of 1 cm in length each (approximately 1 cm^2^ of luminal area), totalizing 36 fragments. The decellularization was performed by two protocols, with 1% sodium dodecyl sulfate (SDS) for 2 hours and 2% sodium deoxycholate (SD) for 1 hour (both under agitation in a Shaker News Brunswick Scientific^®^, at 37°C). The fragments were stored in a refrigerator at 4°C in a sterile solution containing antibiotics and fungicide.

An additional step of exposition to 1% TritonX-100 (TX) for 10 minutes was performed for six scaffolds of each detergent.

Six fragments were exposed to poly-l’lysine and laminin for 30 minutes (each one), and the other six were maintained without other processing steps for each detergent.

### Obtaining of adipose-derived stem cells

For the obtainment of the ASC, 2 g of AT was surgically removed from the interscapular region of the same rabbits and stored in Falcon with N-2-hydroxyethylpiperazine-N-2-ethane sulfonic acid (HEPES) solution containing penicillin, 100 mg/mL streptomycin, and 25 mg/mL amphotericin B (2 mmol/L l-glutamine; Invitrogen™). ASCs were acquired through enzymatic dissociation with type I collagenase (Invitrogen™). Cell culture procedures were done with an initial cell count of 2 × 10^4^ cells/cm^2^, obtained from five adipose tissue fragments. These cells were seeded and expanded in six-well culture plates using Dulbecco’s modified Eagle’s (DMEN), supplemented with 10% fetal bovine serum (FBS), 100 U/mL penicillin, 100 mg/mL streptomycin, 25 mg/mL amphotericin B (2 mmol/L l-glutamine; Invitrogen™), 1% (v/v) minimum essential medium (MEM) essential amino acids solution (Invitrogen™), and 0.5% (v/v) of 10 mM MEM nonessential amino acids solution (Invitrogen™) until reaching the number of cells needed for the experiments. The ASC were phenotypically analyzed through flow cytometry, using CD45, CD44, CD90, and CD11b, and through differentiation techniques in trilineage (StemPro adipogenesis, chondrogenesis, and osteogenesis kits; Invitrogen).

### Cell spreading in the biological scaffold

The ASCs were washed with D-PBS, diluted with the PuraMatrix^®^ peptide hydrogel (BD Biosciences), and 10 µL was pipetted in the lumens of each scaffold. The experiment was conducted in triplicate, maintained in culture for 21 days in M199 growth media supplemented with 10% SFB and growth factors to induce differentiation in the endothelium, 10 ng/mL VEGF, 50 ng/mL ?FGF, 20 ng/mL, and M199 supplemented^20^.

The experiment was composed of 12 groups, each one in triplicate, with 1 cm^2^ of the luminal area on scaffolds:

- group 1: IVC-SDS+1 × 10^5^ ASC;
- group 2: IVC-SDS+1 × 10^6^ ASC;
- group 3: IVC-SDS-TX+1 × 10^5^ ASC;
- group 4: IVC-SDS-TX+1 × 10^6^ ASC;
- group 5: IVC-SDS+P/L+1 × 10^5^ ASC;
- group 6: IVC-SDS+P/L+ 1 × 10^6^ ASC;
- group 7: IVC-SD+1 × 10^5^ ASC;
- group 8: IVC-SD+1 × 10^6^ ASC;
- group 9: IVC-SD+TX+1 × 10^5^ ASC;
- group 10: IVC-SD-TX+1 × 10^6^ ASC;
- group 11: IVC-SD+P/L+ 1 × 10^5^ ASC;
- group 12: IVC-SD+P/L+1 × 10^6^ ASC.

These cell-seeded scaffolds were further incubated for 30 minutes at 37°C with a 5% CO_2_ incubator and a culture medium, which were placed in an appropriate Petri dish (40 × 30 mm). A minimal amount (30 µL) of M199 medium was added to the scaffold to induce the gelling of the solution.

The scaffold spread with the cells was then transported to a 24-well cell culture plate (Corning^®^ Costar^®^ Ultra-Low Attachment Multiple Well Plate) and completed with another 3 mL of the M199 medium supplemented. The culture plates were kept in a humidity-controlled environment, at 37°C, and 5% CO_2_, with M199, increased every two days, for 21 days total.

### Analysis of scaffolding cell colonization

After the conclusion of the experimental culture, the fragments were collected and sectioned into two parts, one of them was cryopreserved in liquid nitrogen to obtain fresh histological slides to undergo immunofluorescence with 4’,6-Diamidine-2’-phenylindole dihydrochloride (DAPI), and the other one was paraffinized for slide preparation and stained in hematoxylin and eosin (HE).

For cell counting in HE, the slides were analyzed under optical microscopy at x100. Each group consisted of triplicates, three slides out of each. All viable cells were counted in five fields in a x100 optical microscope (allowing the count of all cells in fragments), totalizing 15 analysis per group. A histological slide of the freshly frozen material was cut in a cryostat and proceeded to specific immunofluorescence with DAPI to mark the nucleic acid (nuclear) of the ASCs (in blue). A statistical test of comparison and agreement for cell counts by the two forms of histological analysis was performed to ascertain whether processing methods could interfere with cell count analysis.

### Statistical analysis

Statistical analysis of cell counting was done using a generalized linear model (GLM) with Poisson’s distribution and a log link function, followed by Wald’s chi-square test for multiple comparisons.

The Mann-Whitney test was used to perform a comparative analysis between the global cell count determined by each of the two methods of histological analysis. Continuous numerical data are expressed as median (range) according to the data distribution as determined by the Kolmogorov-Smirnov test for normality. The agreement between the cell count determined by the methods of histological analysis was evaluated using interclass coefficient correlation for two-way random effects. For statistical analysis, significant values were considered when p < 0.05 (Software IBM SPSS statistics version 1.0.0.1406).

## Results

The culture of ASC, their characterization, and the obtaining of scaffolds were performed as previously described in methods.

### Histological analysis

#### Hematoxylin & eosin cell counting

**Figure 1:**
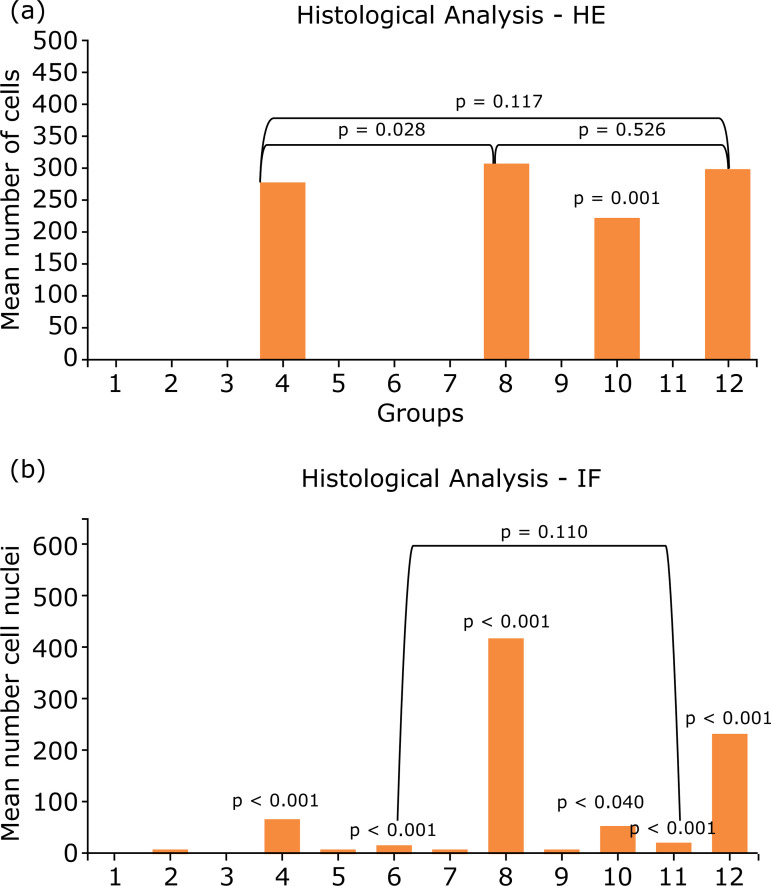
Analysis of histological methods. **(a)** Analysis of cell count in HE slides; **(b)** Analysis of nuclei cell count in immunofluorescence stained with DAPI.

HE: hematoxylin and eosin; IF: immunofluorescence; DAPI: 4’,6-Diamidine-2’-phenylindole dihydrochloride.

**Figure 2:**
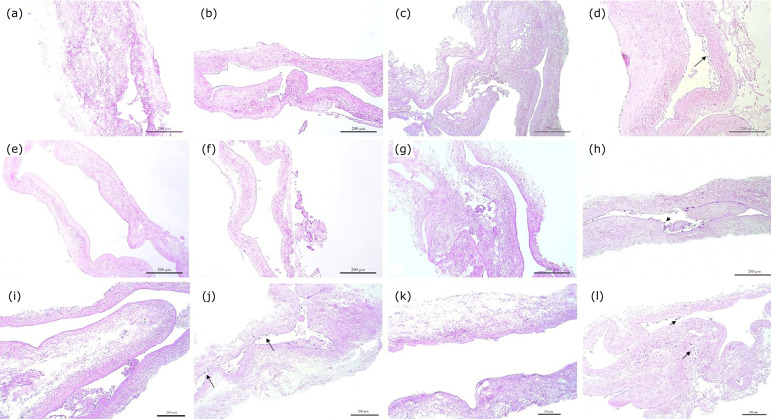
Analysis of cell count (arrows) in hematoxylin and eosin (HE) slides of the groups: **(a)** Group 1 IVC-SDS+1 × 10^5^ ASC; **(b)** Group 2 IVC-SDS+1 × 10^6^ ASC; **(c)** Group 3 IVC-SDS-TX+1 × 10^5^ ASC; **(d)** Group 4 IVC-SDS-TX+1 × 10^6^ ASC; **(e)** Group 5 IVC-SDS+P/L+1 × 10^5^ ASC; **(f)** Group 6 IVC-SDS+P/L+ 1 × 10^6^ ASC; **(g)** Group 7 IVC-SD+1 × 10^5^ ASC; **(h)** Group 8 IVC-SD+1 × 10^6^ ASC; **(i)** Group 9 IVC-SD+TX+1 × 10^5^ ASC; **(j)** Group 10 IVC-SD-TX+1 × 10^6^ ASC; **(k)** Group 11 IVC-SD+P/L+ 1 × 10^5^ ASC; **(l)** Group 12 IVC-SD+P/L+1 × 10^6^ ASC.

Given the histological analysis stained with hematoxylin and eosin (HE), the cell counting showed that, by the GLM, there was a significant difference between the groups (p < 0.001), and, through the Wald chi-square test for multiple comparisons, the groups with the best cell adhesion were: group 8 (IVC-SD-1 × 10^6^ ASC) and group 12 (IVC-SD-P/L-1 × 10^6^ ASC), without a statistic difference between them (p = 0.526), followed by group 4 (IVC-SDS-TX-1 × 10^6^ ASC), which showed statistical lower results than group 8 (SD-1 × 10^6^ ASC) (p = 0.028) and showed no statistical difference to group 12 (IVC-SD-P/L-1 × 10^6^ ASC) (p = 0.117), followed by group 10 (IVC-SD-TX-1 × 10^6^ ASC), which was less than the previous three (p < 0.001). The other groups did not present significant cells, considered null results, and differed significantly from the other groups (p < 0.001). Figures 1 (a) and 2 show these results in detail.

### Immunofluorescence DAPI cell nuclei counting

**Figure 3:**
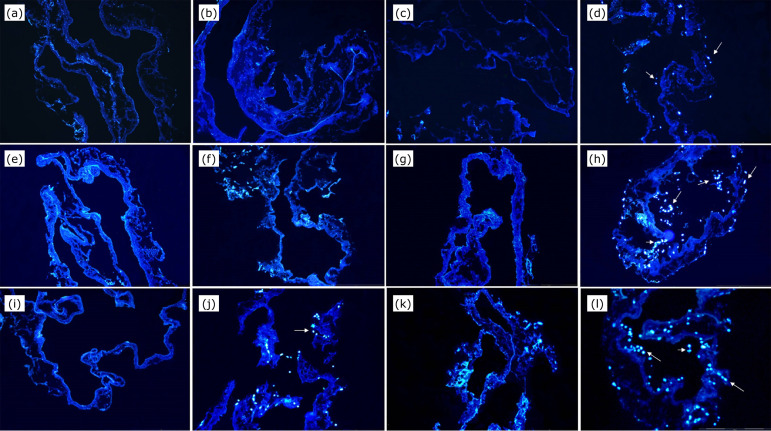
Analysis of nuclei cell count (arrows) in immunofluorescence stained with DAPI slides of the groups: **(a)** Group 1 IVC-SDS+1 × 10^5^ ASC; **(b)** Group 2 IVC-SDS+1 × 10^6^ ASC; **(c)** Group 3 IVC-SDS-TX+1 × 10^5^ ASC; **(d)** Group 4 IVC-SDS-TX+1 × 10^6^ ASC; (e) Group 5 IVC-SDS+P/L+1 × 10^5^ ASC; **(f)** Group 6 IVC-SDS+P/L+ 1 × 10^6^ ASC; **(g)** Group 7 IVC-SD+1 × 10^5^ ASC; **(h)** Group 8 IVC-SD+1 × 10^6^ ASC; **(i)** Group 9 IVC-SD+TX+1 × 10^5^ ASC; **(j)** Group 10 IVC-SD-TX+1 × 10^6^ ASC; **(k)** Group 11 IVC-SD+P/L+ 1 × 10^5^ ASC; **(l)** Group 12 IVC-SD+P/L+1 × 10^6^ ASC.

Given the histological analysis by immunofluorescence (IF), the cellular nuclei counting showed that, by the GLM, there was a significant difference between the groups (p < 0.001), and, through the Wald chi-square test for multiple comparisons, the groups with better cell adhesion were: group 8 IVC-SD-1 × 10^6^ ASC (p < 0.001), followed by group 12 IVC-SD-P/L-1 × 10^6^ ASC (p < 0.001), then by group 4 IVC-SDS-TX-1 × 10^6^ ASC, which showed a statistical difference for the next group (p = 0.04), that was group 10 IVC-SD-TX-1 × 10^6^ ASC. These groups were superior that group 6 IVC-SDS+P/L+1 × 10^6^ ASC and group 11 IVC-SD+P/L+1 × 10^5^ ASC (p < 0.001), but without differences between them (p = 0.110). The remaining groups showed an insignificant number of cells nuclei (mean less than eight per repetition). Figures 1 (b) and 3 show these results.

DAPI: 4’,6-Diamidine-2’-phenylindole dihydrochloride.

### Comparative and concordance analysis of cell counts by the two histological methods

The global cell count determined by IF was higher than the number picked by HE 10.5 (0/820) × 0 (0/640); p = 0.002. The interclass correlation coefficient was 0.315, which represents fair agreement.

## Discussion

Biological scaffolds are a great source of biomaterials, with advantages such as hydrophilicity, low toxicity, and low immunogenicity, in addition to good adhesion and cell multiplication. Therefore, when using biological scaffolding, the native architecture is highly preserved, and the antigen content was removed during the decellularization process^17^.

The scientific literature presents several decellularizing agents and different methods. However, we seek a practical protocol, which has high efficiency and speed, but not only that, one that promotes an enhanced environment for cell adhesion and colonization, specifically for TEBV^13^^,^ 14 ^,^ 16 ^,^ 17 ^-^ 19 ^,^ 21 ^-^ 26.

Our team’s previous experimental studies demonstrated that rabbit’s vena cava could be decellularized while maintaining an excellent structural matrix, therefore serving as a promising scaffold to receive the ASC derived from adipose tissue^14^. It was also determined that the process of decellularization of the vein does not cause significant residual toxicity or loss of essential characteristics of the extracellular matrix^14^. However, cell adhesion and proliferation did not show regularity during the several repetitions of the method, mainly regarding the number of cells. Thus, this experiment was designed to check the possible variables that would lead to the best results: the number of cells, the type of decellularizing agent, or the addition of TritonX-100 or poly-l’lysine + laminin.

Published studies demonstrated that, for a suitable recellularization of a vascular scaffold, it is required at least 1 × 10^6^ cells per cm^2^ and at least two weeks of culture, which was also observed in our experiments with vein scaffolds seeded with 1 × 10^6^ ASC^21^. Studies concluded that the decellularization of veins by the SDS protocol has a well-preserved extracellular matrix, membrane structure, and sufficient strength for a vascular graft^22^^-^ 24. On the other hand, Zhou *et al*.^25^ stated that decellularization with SDS could be harmful to structural and signaling proteins, such as the collagen of some cardiac tissues that remain damaged, despite successfully removing cells. According to our best protocols, SD was observed to be more favorable to seeded cells. Still, the TritonX-100 helped SDS protocol and increased its efficacy, assuming the third position between them^16^^,^ 24 ^,^ 26. The decellularization of tissues with SD eliminates cells, preserving the extracellular matrix, and in this study it presented advantages in cellular interaction, thus making the protocol promising to produce human blood vessel scaffolds for application in TEBV.

Previous studies that used TritonX-100, an anionic detergent, to enhance the decellularizing action of other enzymatic detergents, such as SDS and SD, obtained better results on removing cells from thicker tissues, such as heart valves, in which the enzymatic or osmotic methods alone were ineffective. This association can be beneficial, because it can decrease the loss of extracellular matrix protein, balance repulsive negative ionic charges in the cells, and concomitantly decrease adverse immune response *in vivo* 14 ^,^ 18 ^,^ 25 ^,^ 26. However, ionic detergents (SDS and SD) can solubilize lipids, cytoplasmic membranes, and protein denaturation. Still, some studies point to collagen denaturation and the difficulty of removing the remaining matrix, which negatively influences the scaffolds’ cytocompatibility^16^. This study suggested the best result of recellularization in scaffolds produced by SD protocols.

Poly-l’lysine is commonly used in cell and tissue culture as a fixation factor to enhance cell adhesion through the interaction of the polymer (positive charge) and cells/protein (negative control)^16^^,^ 27. Laminin is a glycoprotein of fundamental importance in helping differentiation, migration, and cell adhesion, acting as a source of protein network, and organizing the extracellular matrix’s formation. Our experiment with SD in association with poly-l’lysine and laminin did not improve the results, characterizing that this protocol does not need any additives to increase cell adhesion^16^^,^ 27.

The comparison between the histological methods with fixation process for making paraffinized blocks and cuts for conventional HE histology was inferior in the detection of the sown cells compared to the method that used fresh frozen material and immunofluorescence, since the cells adhered in the lumen of the scaffolds (still immature and with little extracellular fixation matrix) are more sensitive to the physical processing required to produce paraffinized blocks. Therefore, the method of immunofluorescence with DAPI (with freshly frozen material) showed greater sensitivity in identifying cells, with a small methodological agreement after the statistical tests, following the scientific literature^28^. Thus, for early cell adhesion assays, it is suggested that this method may be advantageous concerning conventional histology for identifying cells.

## Conclusions

This experiment demonstrates that the decellularizing agent and the number of ASC seeded influence the recellularization response of vena cava scaffolds. In this way, it was possible to identify the best protocols that used SD with or without the addition of poly-l’lysine and laminin. In the sequence, it was observed that the addition of TrintonX-100 to the SDS protocol could improve cellular adhesion. Fundamentally, the number of cells applied influenced all protocols progressively. New experiments should be carried out to evaluate the in-vivo behavior of these bioengineered products to determine their functionality.
